# Viral Load Suppression after Enhanced Adherence Counseling and Its Predictors among High Viral Load HIV Seropositive People in North Wollo Zone Public Hospitals, Northeast Ethiopia, 2019: Retrospective Cohort Study

**DOI:** 10.1155/2020/8909232

**Published:** 2020-04-21

**Authors:** Gedefaw Diress, Samuel Dagne, Birhan Alemnew, Seteamlak Adane, Amanuel Addisu

**Affiliations:** ^1^College of Health Sciences, Woldia University, Woldia, Ethiopia; ^2^College of Medicine and Health Science, Bahir Dar University, Bahir Dar, Ethiopia

## Abstract

**Background:**

The World Health Organization currently encourages enhanced adherence counseling for human immunodeficiency virus (HIV) seropositive people with a high viral load count before a treatment switch to the second-line regimen, yet little is known about viral load suppression after the outcome of enhanced adherence counseling. Therefore, this study aimed to assess viral suppression after enhanced adherence counseling sessions and its predictors among high viral load HIV seropositive people.

**Methods:**

Institutional-based retrospective cohort study was conducted among 235 randomly selected HIV seropositive people who were on ART and had a high viral load (>1000 copies/ml) from June 2016 to January 2019. The proportion of viral load suppression after enhanced adherence counseling was determined. Time to completion of counseling sessions and time to second viral load tests were estimated by the Kaplan–Meier curve. Log binomial regression was used to identify predictors of viral re-suppression after enhanced adherence counseling sessions.

**Result:**

The overall viral load suppression after enhanced adherence counseling was 66.4% (60.0–72.4). The median time to start adherence counseling session after high viral load detected date was 8 weeks (IQR 4–8 weeks), and the median time to complete the counseling session was 13 weeks (IQR 8–25 weeks). The probability of viral load suppression was higher among females (ARR = 1.2, 95% CI: 1.02–1.19) and higher educational status (ARR = 1.7, 95% CI: 1.25–2.16). The probability of viral load suppression was lower among people who had 36–59 months duration on ART (ARR = 0.35, 95% CI: 0.130–0.9491) and people who had > 10,000 baseline viral load count (ARR = 0.44, 95% CI: 0.28–0.71).

**Conclusion:**

This study showed that viral suppression after enhanced adherence counseling was near to the WHO target (70%) but highlights gaps in time to enrolment into counseling session, timely completion of counseling session, and repeat viral load testing after completing the session.

## 1. Introduction

In the mid-2017, 20.9 million people living with HIV were taking antiretroviral therapy (ART) globally [1] and monitoring of people on ART is crucial to ascertain successful treatment, identify adherence challenges, and determine whether ART regimens should be switched in cases of treatment failure [2]. The World Health Organization (WHO) recommended the use of viral load testing as the gold standard to monitor the patient's responses to ART [2]. The three 90s (90-90-90) strategy which was developed by UNAIDS clearly states that 90% of all people on ART should have a suppressed viral load [3].

Initial viral load testing in people with HIV should be after 6 months of initiating ART and every 12 months thereafter routinely. Besides, targeted viral load testing was offered for suspected clinical or immunological failure [4]. The result of a viral load test could be unsuppressed (viral load count greater than 1000 copies/ml), which implies that HIV is not controlled by the current ART regimen. Past evidence showed that viral suppression at the first test is much lower than expected and viral replication continues to be a major challenge among patients living with HIV. Patients who have unsuppressed viral load were found to have an increased risk of clinical progression to acquired immunodeficiency syndrome (AIDS) and mortality when compared with patients with a complete virologic response [5].

Since poor adherence is the most common reason for treatment failure [6–8], the WHO recommends enhanced adherence counseling sessions for 3–6 months for people with high viral load count before diagnosing first-line treatment failure. Enhanced adherence counseling (EAC) is a continual and repeated process that involves a structured assessment of the current level of adherence, explore the specific barriers the patient must overcome, assisting patients to identify solutions, and address barriers and develop an individualized adherence interventional plan which improve viral load suppression and reduces subsequent treatment failure [9].

Performing repeated viral load test is recommended by the WHO for all people with unsuppressed viral load (>1000 copies/ml). If the patient complete 3 EAC monthly sessions each one month apart, the client is perfectly adherent for 3–6 months and there is no opportunistic infection during the 3–6 months [10]. Even though the recommended time to complete the three EAC sessions was 90 days, the previous study conducted in Uganda showed that only 37% of patients complete the sessions in the recommended time interval. About 16% of patients who enrolled to EAC received only one or two EAC sessions [11], which implies poor handling of patients with unsuppressed viral load. This triggers us to conduct this study for estimation of time of completion of EAC session and time to second viral load test in Ethiopia.

According to the WHO estimation, up to 70 % of patients with initial high viral load count (greater than 1000 copies/ml) achieved virologic suppression after 3–6 enhanced adherence counseling session [10], but only 23% of patients achieved viral load suppression after enhanced adherence counseling in Uganda [11].

In March 2016, Ethiopia Federal Ministry of Health initiated routine viral load testing for all HIV seropositive people with the goal of all eligible patients receiving at least one viral load test every year [10]. In Ethiopia, a total of 128,615 (54% of total HIV seropositive people on ART) viral load tests were performed from March 2016 to January 2019. Viral load suppression at the first viral load test was reported in 73% of pediatric patients and 83.3% of adult patients. This implies in Ethiopia 27% of pediatrics and 17% of adults required enhanced adherence counseling and repeat viral load testing after 3–6 months [12]. In North Wollo Governmental Hospitals, from 6245 currently on ART patients, around 4012 viral load tests were performed between the years 2016 and 2019. High viral load copies (>1000 copies/ml) at the first test were reported in 568 patients [13].

Furthermore, to our knowledge, no research has been done in Ethiopia which assessed viral load suppression after EAC sessions among high viral load HIV seropositive people who are on ART. And the finding of this research will help Ethiopian Ministry of Health and other stakeholders to assess the progress in the implementation of the national guidelines focus on EAC and to improve the patient's quality of life. Therefore, this study aimed to assess viral load suppression after EAC, estimate time to completion of the EAC session, estimate time to second viral load test, and identify the predictors of viral load suppression among high viral load HIV seropositive people on ART.

## 2. Materials and Methods

### 2.1. Study Setting

This study was conducted at three public hospitals which provide ART service in North Wollo Zone [14]. All the three hospitals started conducting routine viral load testing for all HIV infected people from June 2016 onwards as per the national guidelines. Those with viral load >1000 copies/ml are referred for enrolment in EAC. Enhanced adherence counseling consists of three sessions done on a monthly basis. After three EAC sessions, each client is assessed for adherence and a repeat viral load test is performed. If the viral load is suppressed (≤ 1000 copies/ml of blood), the client is continued on the same ART regimen. On the other hand, if the repeat viral load is greater than 1000 copies/ml despite good adherence to therapy, the client is switched to the second-line ART regimen.

### 2.2. Study Design and Period

An institutional-based retrospective cohort study was conducted from March to May 2019.

### 2.3. Population

#### 2.3.1. Source Population

All HIV infected people on the first-line regimen who had viral loads >1000 copies/ml after 6 months on ART between June 2016 and January 2019 at three public hospitals of North Wollo Zone.

#### 2.3.2. Study Population

All HIV infected people with viral load greater than 1000 copies/ml at the first test and had at least one repeated viral load result after complete enhanced adherence counseling session in Governmental Hospitals of North Wollo Zone.

### 2.4. Eligibility Criteria

#### 2.4.1. Inclusion Criteria

All HIV infected people with documented viral load results greater than 1000 copies/ml.

#### 2.4.2. Exclusion Criteria

Clients who had a follow-up of fewer than 6 months, patients who did not start EAC session, and clients who had no second viral load result were excluded.

### 2.5. Sample Size Calculation

The required sample size was calculated by Epi-Info version 7 with the following assumption:

1. 95% CI
2. 80% power
3. The percentage of outcome (viral suppression) in the exposed group (female) is 10.3% by selecting sex (a variable which result in large sample size) as exposure variable from a study performed in Uganda.

The calculated sample size was 213. By adding 10% for incomplete data, the final sample size reached 235.

### 2.6. Sampling Procedure

All three public hospitals were included in the study, and the total sample size was allocated proportionally among each hospital based on the number of patients with a viral load count of greater than 1000 copies/ml. The study participants were selected through simple random sampling technique from a viral load registration book.

### 2.7. Data Collection Tools and Procedure

Data were collected from patient's chart, enhanced adherence counseling sheet, viral load registration book, and laboratory request using a pretested structured checklist. Accordingly, all charts containing detailed information about patients who were on ART were reviewed. When there were incomplete data, the data collectors have tried to get from different data sources (patient's chart and follow-up form). If clinical parameters and laboratory results (CD4 count and WHO clinical stage) were not found at the start of enhanced adherence counseling sessions, the data which were most recent to the starting date of enhanced adherence counseling sessions were considered as baseline data. Six BSc degree nurse as data collectors and three ART data managers as supervisor were participated in the data collection process.

### 2.8. Variables

The main outcome of interest was viral suppression (suppressed/unsuppressed). Explanatory variables were age, the current ART regimen, viral load count at the start of EAC session, sex, WHO clinical stage, baseline CD4 count, nutritional status, residence, opportunistic infections (OIs), functional status, isoniazid preventive therapy status, cotrimoxazole preventive therapy (CPT) status, duration on ART (determined using date of ART initiation and date of ART clinic visit), and level of ART adherence. Baseline means at the time of high viral load determination (recent to the study). Opportunistic infection is infections that occur more frequently or more severe in people with HIV than in people with healthy immune systems, and it is used as criteria for the WHO clinical staging.

### 2.9. Measurements

Viral load was determined through real-time polymerase chain reaction (PCR) assay. Viral load after EAC was classified as suppressed or unsuppressed based on 2018 Ethiopian national guidelines for comprehensive HIV prevention, care, and treatment. Viral load suppression is considered when viral load is less than or equal to 1000 copies/ml after EAC, and high viral load is defined when a client is found to have a viral load of >1000 copies/ml on a routine or need-based viral load test. Level of ART adherence was classified as good/fair/poor based on the percentage of ARV drug taken (good ART adherence defined as 95% or greater of doses taken as prescribed, fair adherence defined as 85–94% of doses taken as prescribed, and poor adherence defined as less than 85% of doses taken as prescribed) [15].

### 2.10. Data Quality Management

Data quality was assured through designing a proper data collection material. Two days long training was given for both data collectors and supervisors on the objective of the study, data extraction, and recording from patient's chart and registration books. During the data collection period, a supervisor was assigned to make sure that there were no missed data. The overall activities were controlled by the principal investigator of the study. By taking 10% of participants randomly from Dessie Referral Hospital, pretest was performed to assess the reliability and consistency of the data collection tool. The data were entered by double entry, and at the end of data entry, data cleaning was carried out using frequency, cross tabulation, sorting, and listing to check missed value and outliers.

### 2.11. Data Analysis Procedure

Data were entered into Epi data version 3.1 and analyzed using Stata version 14. Descriptive statistics, including mean and frequencies, were used to describe characteristics of the study participants. Viral load suppression after EAC sessions was presented as proportion. Time to completion of EAC session and time to second viral load test was presented by the Kaplan–Meier curve (survival function). Log binomial regression model was used to identify predictors of viral load suppression. We preferred log binomial regression over logistic regression model in this study to report adjusted risk ratio because the incidence of outcome was greater than 10%.

### 2.12. Ethical Consideration

Ethical clearance was obtained from the Institutional Review Committee of Woldia University. Since it was a retrospective analysis of the identified data, a waiver of consent was obtained. Even though most of the data were collected from the patient chart and follow-up card, a written patient consent form without any personal identifiers was prepared. The recorded data were not accessed by a third person except by the investigators in order to kept confidentiality.

## 3. Result

### 3.1. Baseline Characteristics of Study Participants

A total of 235 participants were included in the study, of whom 121 (51.5%) were female. The mean age of study participants was 33.1 (±12.49SD). Majority (109 (46.4%)) of participants were married, and around 36% of participants have completed the primary educational level. At baseline, most of the participants were staged in WHO clinical stage 1 (90.6%), had a CD4 count above or equal to 500 cells/*μ*l (42.3%), and 83% of participants had good ART adherence level at the start of EAC sessions. From all participants, 105(44.7%) had a viral load count between 1,000 and 5,000 copies/ml when enrolled to EAC sessions (Table 1).

### 3.2. Proportion of Viral Load Suppression after EAC

From a total of 235 participants, 66.4% (60.0–72.4) of participants had viral load suppression after enhanced adherence counseling intervention. From all viral load suppressed participants, 53.2% were male and 46.1% were married. Majority (42.8%) of viral load suppressed participants had primary educational status, and approximately 73% of viral load suppressed participants were at the efavirenz (EFV)based ART regimen. Almost half of viral load suppressed patients had 1000–5000 viral load copies/ml at the start of EAC sessions, and 92% of viral load patients had good ART adherence level at baseline (Table 2).

### 3.3. Factors Associated with Viral Load Suppression

On bivariate analysis gender, educational status, residence, baseline CD4 count, first viral load count, and baseline adherence level were significantly associated with viral load suppression. On multivariable analysis gender, educational status, duration on ART, and baseline viral load count greater than 1000 copies/ml were significant predictors of viral load suppression (Table 3).

Females were 1.2 times more likely to have viral load suppression as compared to male participants (ARR = 1.2, 95% CI: 1.02–1.19). Primary or secondary and above educational status was associated with the risk of virologic suppression compared to those who cannot read and write (ARR = 1.4, 95% CI: 1.03–1.84, and ARR = 1.7, 95% CI: 1.25–2.16, respectively). More than 12 months on ART was associated with decreased probability of suppression as compared to less than 12 months on ART (13–35 months, ARR = 0.11, 95% CI: 0.03–0.38; 36–59 months, ARR = 0.35, 95% CI: 0.130–0.9491). But more than 60 months on ART was not significantly associated with viral load suppression. The probability of viral load suppression was 7% lower for participants who had 5001–10,000 copies/ml viral load count as compared to those who had 1000–5000 copies/ml (ARR = 0.93, 95% CI: 0.87–0.99). Similarly, the probability of viral load suppression was 56% lower for participants who had viral load count greater than 10,000 compared to those who had 1,000–5,000 copies/ml (ARR = 0.44, 95% CI: 0.28–0.71) (Table 3).

### 3.4. Median Time to the Start of EAC Session after High Viral Load Detected

The median time to the start of the EAC sessions after high viral load (>1000 copies/ml) detected date was 8 weeks (IQR 4–8 weeks). Significant number of participants (78 (33.2%)) have started EAC sessions after 4–8 weeks of high viral load detection and around 13% of participants did not start the EAC sessions up to 3 months. The median time to take a second viral load test after complete enhanced adherence counseling session was 4 weeks (IQR 0–6 weeks). Half of the participants had a second viral load test within 2 weeks of completing the EAC session, but 28 (12%) participants did not take the second viral load test for more than 8 weeks (Table 4).

### 3.5. Time to Complete Enhanced Adherence Counseling (EAC) Sessions

**Figure 1:**
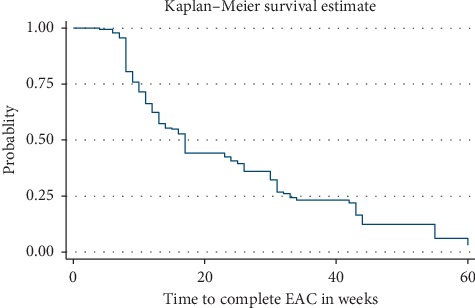
Kaplan–Meier survival curves showing failure experiences of patients who completed EAC.

**Figure 2:**
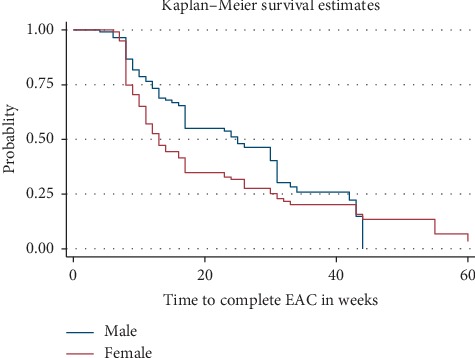
Kaplan–Meier survival curves showing failure experiences of patients who completed EAC by sex.

The median time to complete the EAC session was 17 weeks (IQR 10–33 weeks) (Figure 1) and differs by gender. It was 25 weeks in males and 13 weeks in females (Figure 2). Similarly, the median time to complete EAC was 13 weeks in urban and 26 weeks in rural. In this study, only 46.8% of participants have completed the EAC sessions within 3 months. Nearly one-third (27.2%) of high viral load patients completed the EAC sessions after 6 months of initiation of counseling sessions (Table 4).

## 4. Discussion

This is one of the first studies in Ethiopia which assessed the outcome of the EAC program on HIV seropositive people with high viral load count. Our findings suggest that the overall viral load suppression after enhanced adherence counseling sessions was 66.4% (60.0–72.4). From all HIV seropositive with high viral load who were eligible for EAC, two-thirds of patients had viral load suppression (viral load less than 1000 copies/ml) after the EAC sessions conducted for 3–6 months. This re-suppression rate is near to the WHO target (70%) [3]. But it is much higher than the viral suppression rates reported in Zimbabwe [16] and Uganda [11]. Most importantly, the current finding support WHO recommendations that suspected virologic failure (viral load count>1,000 copies/*µ*l at the first test) should be addressed by enhanced adherence counseling as well as repeat measurement before consideration of treatment switch to a second-line drug [3, 10]. Thus, enhanced adherence counseling interventions can preserve the first-line treatment regimen. This could decrease health care costs and the transmission of resistant strains from the newly infected people.

In the current study, there is evidence on delay between ascertaining high viral load count (date of high viral load) and initiating EAC sessions. The result showed that one-third of individuals started the EAC session after 2 months of high viral load detected. This suggests the poor management of high viral load patients which might affect timely detection of treatment failure.

In this study, the EAC sessions were not completed within the recommended time. Of those with high viral loads, only 46.8% of participants completed their EAC session within the recommended time which is 12 weeks. This might be due to long travel distance to health facility because large proportion of participants in the current study were from rural residence. Previous studies also showed that long travel distance and lack of money for traveling to health institution affect ART adherence and follow-up visit within the recommended time [17, 18]. Besides, the median time to complete the enhanced adherence counseling session was greater than 24 weeks in men, which is very far away from the suggested time [3, 10, 12]. This finding implies that some groups of people (men and rural) did not complete the counseling session within the accepted time range, which may lead to unnecessary delays in diagnosis of virologic failure (ART-resistance) and switch to the second-line ART regimen. Delaying ART switch for patients with resistance increases the risk of sexual transmission of ART-resistant strains [19–22], as well as the possibility of subsequent failure on second-line therapy in delays [9, 11, 20, 21]. The healthcare provider must support rural community to complete the EAC session on time.

Being female was significantly associated with viral suppression. This could be due to the timely completion of their EAC session, which might have contributed to better adherence and response to ART therapy in women [20, 23–25]. Besides, in the current study, women had lower baseline viral load levels than men, which might contribute to better viral suppression. But the gender difference in viral suppression rate should be studied further.

Educational status was another factor which was independently associated with viral suppression. Those having primary education and above had high probability of viral load suppression when compared to those who cannot read and write. This is consistent with what has been found in previous evidence, which showed that there was a significant difference in viral suppression rate among different educational levels (67% in incomplete primary education versus 82–87% in individuals with primary education and above) [26]. This could be due to that educated people typically engage in healthier behaviors including ART drug adherence and better understand information during enhanced adherence counseling session [27].

Duration on ART was significantly associated with viral load suppression. Patients who have taken ART for about 13–59 months were less likely to have viral suppression compared to those who have taken ART drug for less than 12 months. This could be associated with destruction of CD4 cells over time. An individual who had greater than or equal to 5,000 copies/ml initial viral load levels were less likely to develop viral load suppression. This finding is supported by previous studies which showed that initial viral load levels may be a good determinant of viral load suppression in patients with high viral load [16, 28].

The major strength of the study was inclusion of all public hospitals in North Wollo Zone and inclusion of all high viral load patients for the last 2 years. The data were collected from the patient chart, follow-up chart, and high viral load registration books, which are the primary level of documentation of the patient information in the country. The key weakness of this study is that data were collected by documentary review, and hence, the analysis and interpretation of the data are restricted to only those variables that are captured in the patient records. Some of the important variables, such as wealth index, distance to health institutions, and substance use, which could have played a major role in initial viral load testing, enrolment for EAC, repeat viral load testing, and viral suppression, were not accessible. In this study, there could have been selection bias arising from the fact that the samples with recorded second viral load could have been obtained from individuals who had regular follow-up or that patients who did not have regular follow-up/drop out patients could have been more likely to be nonsuppressed.

## 5. Conclusion

In this study, about 66.4% of the patients had viral load suppression at 3 months or later. The factors that were statistically associated with viral load suppression on repeat testing at 3 or more months were gender, educational status, duration on ART, and initial viral load count. Results highlight the importance of education in viral load suppression rate. The study shows gaps in time to enrolment into EAC and repeat viral load testing after complete EAC. The explanations for these gaps need to be assessed in future research studies.

## Figures and Tables

**Table 1:** Sociodemographic and clinical characteristics of high viral load HIV seropositive people from June 2016 and January 2019, North Wollo Zone, Ethiopia.

Variables	Category	Frequency	Percentage (%)
Age (years)	<10	9	3.8
	10–19	29	12.4
	20–29	53	22.6
	30–39	60	25.5
	≥40	84	35.7
Sex	Male	114	48.5
	Female	121	51.5
Marital status	Single	65	27.7
	Married	109	46.4
	Widowed	24	10.2
	Divorce	37	15.7
Religion	Orthodox	166	70.6
	Muslim	37	15.7
	Protestant	32	13.7
Educational status	Cannot read and write	61	25.9
	Primary	84	35.8
	Secondary and above	90	38.3
Residence	Urban	132	56.2
	Rural	103	43.8
Type of hospital	General hospital	157	66.8
	Primary hospital	78	33.2
ART regimen	NVP-based regimen	63	26.8
	EFV-based regimen	172	73.2
Duration of ART	<12 months	60	25.5
	12–24 months	48	20.4
	25–59 months	108	46.0
	≥5 years	19	8.1
Adherence level	Good	195	83.0
	Fair	10	4.3
	Poor	30	12.8
WHO stage	Stage 1	213	90.7
	Stage 2 or 3	22	9.3
Functional status	Working	200	85.1
	Ambulatory	30	12.8
	Bedridden	5	2.1
Baseline CD4	<200	93	39.6
	201–500	42	17.9
	≥500	100	42.5
INH eligibility	Yes	165	70.2
	No	70	29.8
Baseline viral load count	1,000–4,900	105	44.7
	5,000–10,000	90	38.3
	≥10,000	40	17.0

**Table 2:** Results showing relationship between baseline characteristics and viral load re-suppression among study participants.

Variables		Viral load		*p* value
		Suppressed (*n* = 156) (%)	Nonsuppressed (*n* = 79) (%)	
Age	<10	6 (3.9)	3 (3.8)	≤0.001
	10–19	26 (16.7)	3 (3.8)	
	20–29	28 (18.0)	25 (31.7)	
	30–39	32 (20.4)	28 (35.4)	
	≥40	64 (41.0)	20 (25.3)	
Sex	Male	63 (53.2)	51 (51.8)	≤0.001
	Female	93 (46.8)	28 (48.2)	
Marital status	Single	45 (29.6)	20 (24.1)	0.120
	Married	70 (46.1)	39 (47.0)	
	Widowed	12 (7.9)	12 (14.5)	
	Divorce	25 (16.4)	12 (14.4)	
Educational status	Cannot read and write	57 (37.5)	39 (47.0)	0.03
	Primary	65 (42.8)	30 (36.1)	
	Secondary and above	24 (15.8)	13 (15.7)	
Residence	Urban	65 (42.8)	34 (41.0)	<0.001
	Rural	87 (57.2)	49 (59.0)	
Type of hospital	General hospital	103 (67.8)	54 (65.1)	0.412
	Primary hospital	49 (32.2)	29 (34.9)	
ART regimen	NVP-based regimen	40 (26.9)	23 (27.7)	0.571
	EFV-based regimen	111 (73.1)	57 (72.3)	
Duration of ART	≤12 months	42 (26.9)	18 (22.8)	0.037
	13–35 months	26 (16.7)	22 (27.9)	
	36–59 months	72 (46.2)	36 (45.6)	
	≥60 months	16 (10.3)	3 (3.8)	
First viral load	1000–5000	79 (50.6)	26 (32.9)	0.011
	5001–10,000	65 (41.7)	25 (31.6)	
	≥10000	12 (7.7)	28 (35.4)	
Baseline CD4	<200	53 (34.0)	40 (50.6)	0.641
	201–500	25 (16.0)	17 (21.5)	
	≥500	78 (50.0)	22 (27.9)	
Adherence level	Good	139 (91.5)	56 (67.5)	0.210
	Fair	3 (2.0)	7 (8.4)	
	Poor	10 (6.5)	20 (24.1)	
WHO stage	Stage 1	139 (91.5)	74 (89.2)	0.012
	Stage 2 or 3	13 (8.6)	7 (8.4)	

ART = antiretroviral therapy; EFV = efavirenz; NVP=nevirapine; WHO = World Health Organization.

**Table 3:** Baseline demographic and clinical characteristics associated with viral load suppression among high viral load HIV infected people after enhanced adherence counselling (EAC) session between June 2016 and January 2019, Ethiopia.

Variables	Category	CRR (95% CI)	ARR (95% CI)	*p* value
Sex	Male	1	1	<0.001
	Female	1.39 (1.148–1.685)^*∗*^	1.18 (1.017–1.192)	

Educational status	Cannot read and write	1		
	Primary	1.62 (1.171–2.241)^*∗*^	1.38 (1.032–1.841)	0.030
	Secondary and above	1.87 (1.378–2.556)^*∗*^	1.65 (1.253–2.164)	<0.001

Residence	Urban	1	1	
	Rural	0.59 (0.472–0.730)^*∗*^	0.66 (0.544–1.805)	0.301

Duration of ART	≤12 months	1	1	
	13–35 months	0.77 (0.568–1.053)	0.11 (0.031–0.379)	0.014
	36–59 months	0.95 (0.770–1.178)	0.35 (0.130–0.949)	0.023
	≥60 months	1.20 (0.932–1.553)	0.40 (0.738–2.217)	0.147

First viral load	1000–5000	1	1	
	5001–10,000	0.96 (0.811–1.136)	0.93 (0.871–0.989)	0.021
	≥10000	0.40 (0.245–0.648)^*∗*^	0.44 (0.277–0.701)	<0.001

Baseline CD4	<200	1	1	
	201–500	1.04 (0.769–1.418)	0.98 (0.985–1.986)	0.101
	≥500	1.37 (1.115–1.680)^*∗*^	0.83 (0.467–1.387)	0.081

Adherence level	Good	1		
	Fair	0.08 (0.012–0.521)^*∗*^	0.12 (0.092–1.021)	0.215
	Poor	0.18 (0.060–0.531)^*∗*^	0.54 (0.240–1.124)	0.131

WHO stage	Stage 1	1	1	1
	Stage 2 or 3	1.03 (0.762–1.392)	1.20 (0.845–1.352)	0.801

CRR = crude risk ratio; ARR = adjusted risk ratio; ART = antiretroviral therapy; WHO=World Health Organization. ^*∗*^ = *p* < 0.05.

**Table 4:** Time from high viral detected to EAC session start and time from EAC complete to repeated viral load.

Category	Subcategory	Frequency	Percentage
Time from high viral load detected to EAC session start	Median (IQR)	8 (4–10 weeks)	—
	<2 weeks	36	15.3
	2–4 weeks	35	14.9
	4–8 weeks	78	33.2
	8–12 weeks	56	23.8
	≥12 weeks	30	12.8

Time to complete EAC sessions	≥ 12 weeks	110	46.8
	13–24 weeks	61	26.0
	≥25 weeks	64	27.2

Time from EAC session complete to repeat viral load	Median (IQR)	4 (0–6 weeks)	—
	<2 week	117	49.8
	2–4 weeks	51	21.7
	4–8 weeks	39	16.6
	8–12 weeks	10	4.3
	≥12 weeks	18	7.7

## Data Availability

The dataset used and analyzed for the study is available from the corresponding author upon reasonable request (gedefawdiress@gmail.com).

## Abbreviations

AIDS: Acquired immunodeficiency syndrome
ART: Antiretroviral therapy
BMI: Body mass index
EAC: Enhanced adherence counseling
HIV: Human immunodeficiency virus
VL: Viral load
WHO: World Health Organization.
